# Supplementary material for: Predicting discrete-time bifurcations with deep learning
Source: Nat Commun. 2023 Oct 10;14:6331. doi: 10.1038/s41467-023-42020-z (PMC10564974; doi:10.1038/s41467-023-42020-z)
Supplement: Supplementary file 1 — Supplementary Information [file 41467_2023_42020_MOESM1_ESM.pdf]

Predicting discrete-time bifurcations with deep learning —Supplemental Material—

Thomas M. Bury[1], Daniel Dylewsky[2], Chris T. Bauch[2], Madhur Anand[3], Leon Glass[1], Alvin Shrier[1*], and Gil Bub[1*]

[1] *Department of Physiology, McGill University, 3655 Promenade Sir William Osler, Montreal, Canada*
[2] *Department of Applied Mathematics, University of Waterloo, Waterloo, Canada*
[3] *School of Environmental Sciences, University of Guelph, Guelph, Canada*
[*] *Joint last authors*

Supplementary Note 1

A discrete-time dynamical system has the form

$$\vec{x}_{t+1} = f(\vec{x}_t) \tag{1}$$

where $\vec{x}_t$ is a vector representing the state of the system and f is a function that maps to the next state. The system is stable about a steady state $\vec{x}_*$ if all eigenvalues (λ) of the Jacobian matrix (gradient of f at $\vec{x}_*$) have a magnitude less than one i.e. they lie within the unit circle on the complex plane (depicted in gray). A local bifurcation occurs when one or a pair of these eigenvalues (the dominant eigenvalue(s)) crosses the unit circle. The type of bifurcation depends on where the unit circle is crossed. A bifurcation is said to be of codimension one if it occurs upon changing only a single parameter of the system.

There are five types of local, discrete-time bifurcations of codimension one: (a) period-doubling; (b) Neimark-Sacker; (c) fold; (d) transcritical; (e) pitchfork. Bifurcation diagrams are shown in the panels below. The x-axis represents the bifurcation parameter and the y-axis a state variable. Solid lines show stable states or limit cycles, and dashed lines show unstable states. Arrows indicate direction of motion at different points in state space. The white points on the unit circle show the value of the dominant eigenvalue(s) at the bifurcation. The Neimark-Sacker bifurcation has a pair of complex conjugate eigenvalues, the angle (θ) being the frequency of oscillations at the bifurcation.

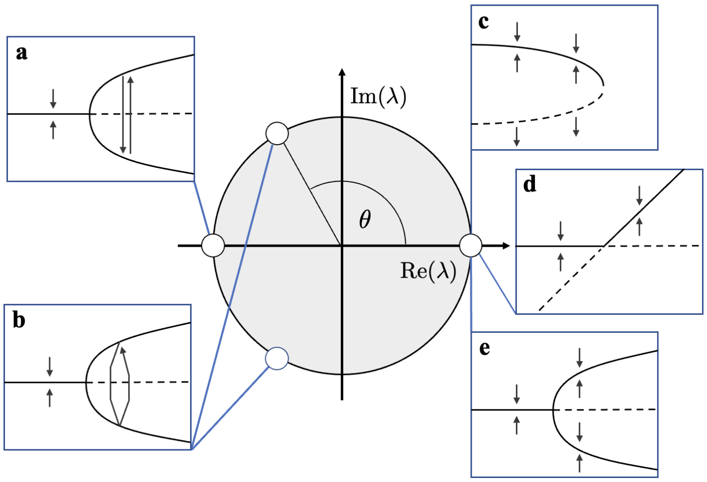

Supplementary Figures

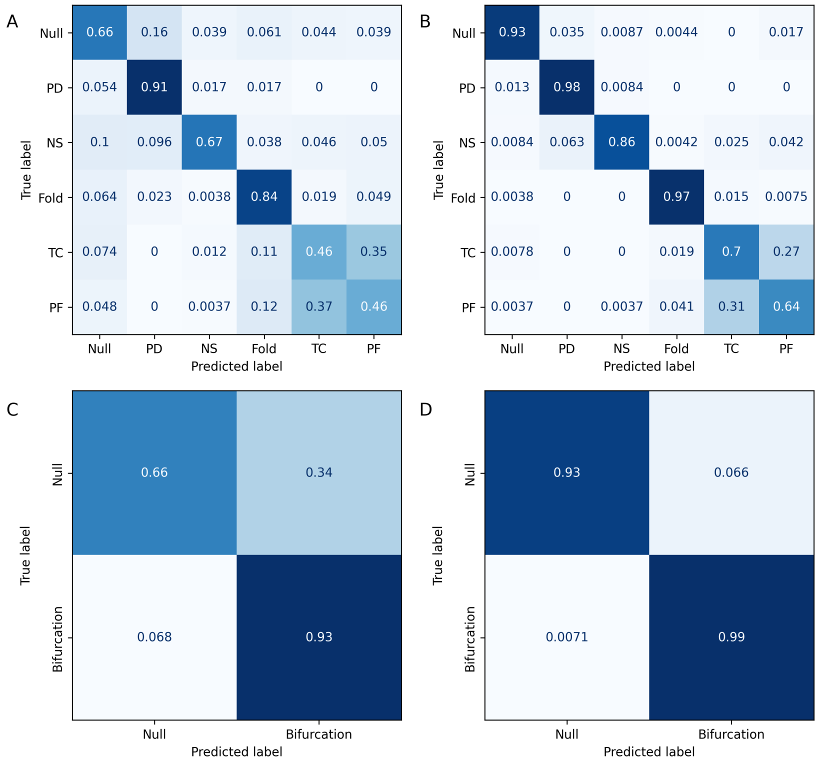

Figure S1: Confusion matrices summarising the performance of Classifier 1 and Classifier 2 on their respective test sets for the multi-class and binary classification problem. Cell values show (row-)normalised classification rates for each class. (A) Classifier 1 on the multi-class classification problem obtains an F1 score of 0.66. (B) Classifier 2 on the multi-class classification problem obtains an F1 score of 0.85. (C) Classifier 1 on the binary classification problem obtains an F1 score of 0.79. (D) Classifier 2 on the binary classification problem obtains an F1 score of 0.97. PD: period-doubling. NS: Neimark-Sacker. TC: transcritical. PF: pitchfork.

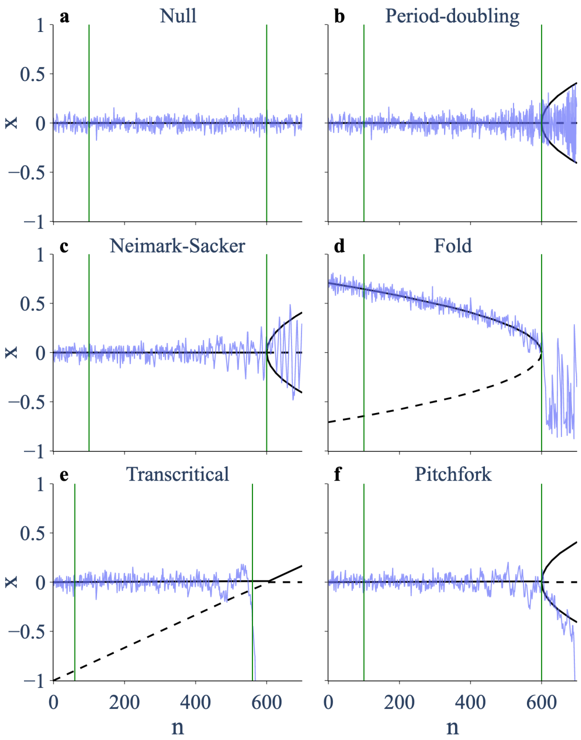

Figure S2: Sample simulations and corresponding bifurcation diagrams for each class in the training data. Panels show the trajectory of x_n (blue), the corresponding bifurcation diagram (black) and the section used for training in each case (between the green vertical lines). The six possible classes are (a) null, (b) period-doubling, (c) Neimark-Sacker, (d) fold, (e) transcritical, and (f) pitchfork. These samples show supercritical versions of the period-doubling, Neimark-Sacker and pitchfork bifurcations, though subcritical examples are also present in the training data. The end time for the extracted data is taken as the time the bifurcation is crossed ($t = 600$) or the first time the deviation from equilibrium reaches ten times the noise amplitude, whichever comes first.

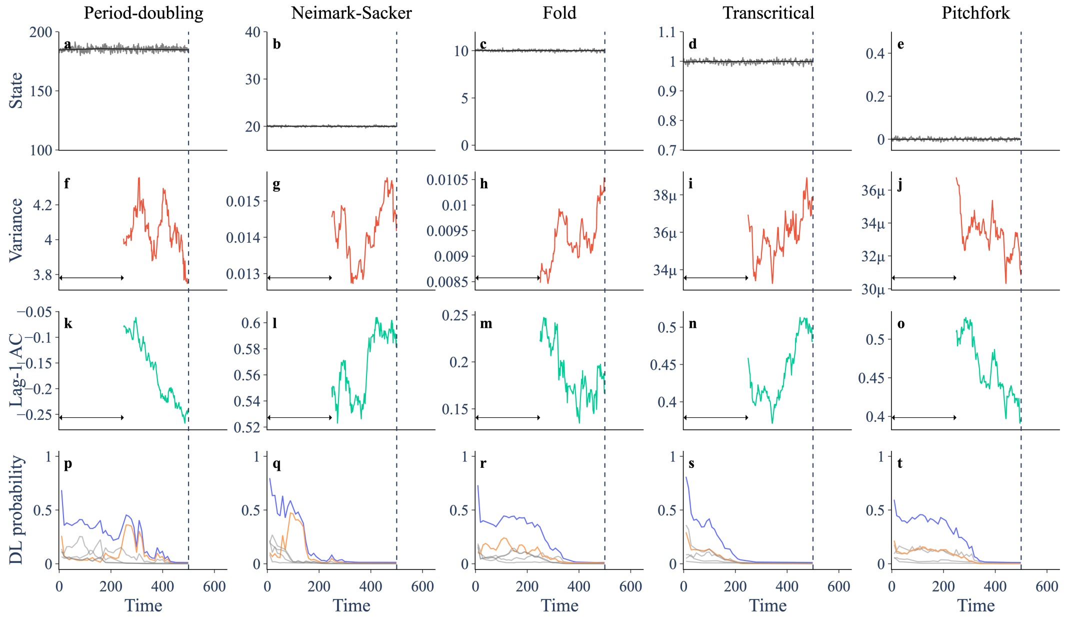

Figure S3: Trends in indicators for null simulations of the theoretical models. (a-e) Trajectory (gray) and smoothing (black) for null simulations of the theoretical model with the period-doubling, Neimark-Sacker, fold, transcritical and pitchfork bifurcation, respectively. Residuals from the smoothing are used to compute EWS. (f-j) Variance of residuals, computed over a rolling window (arrow) of size 0.5 times the length of the pre-transition data. (k-o) Lag-1 autocorrelation of residuals. (p-t) Probabilities assigned by the deep learning (DL) classifier. Orange line shows probability assigned to the bifurcation in the model. Gray lines show probabilities assigned to the other bifurcations. Blue line shows the sum of the five bifurcation probabilities.

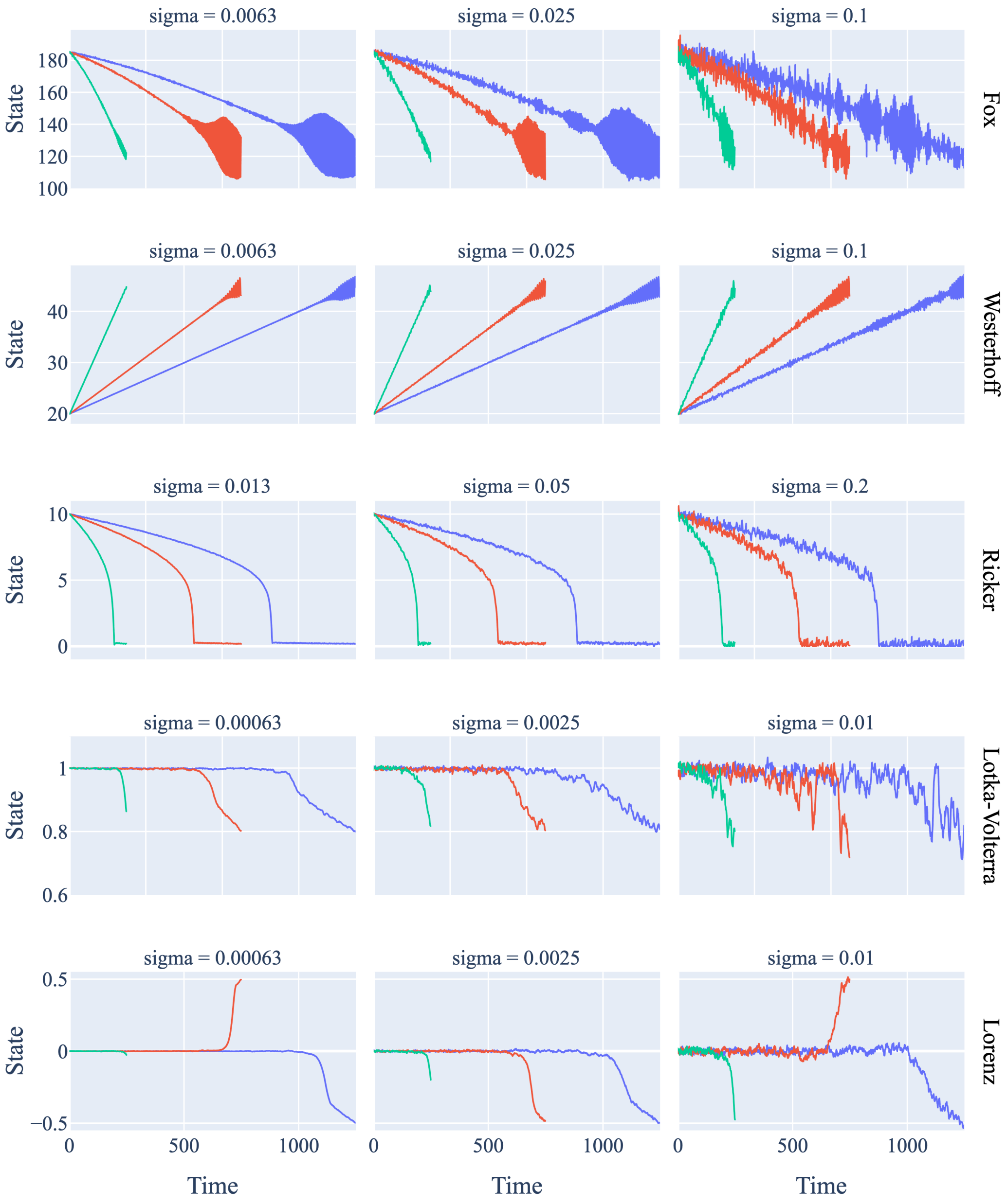

Figure S4: Sample simulations of each theoretical model approaching a bifurcation under a range of noise amplitudes (sigma) and rates of forcing (colour). The rates of forcing are chosen such that the bifurcation is crossed after 100 (green), 300 (red) and 500 (blue) units of time. The Fox model goes through a period-doubling bifurcation, the Westerhoff model a Neimark-Sacker bifurcation, the Ricker model a fold bifurcation, the Lotka-Volterra model a transcritical bifurcation, and the Lorenz model a pitchfork bifurcation. Model parameters are provided in Methods.

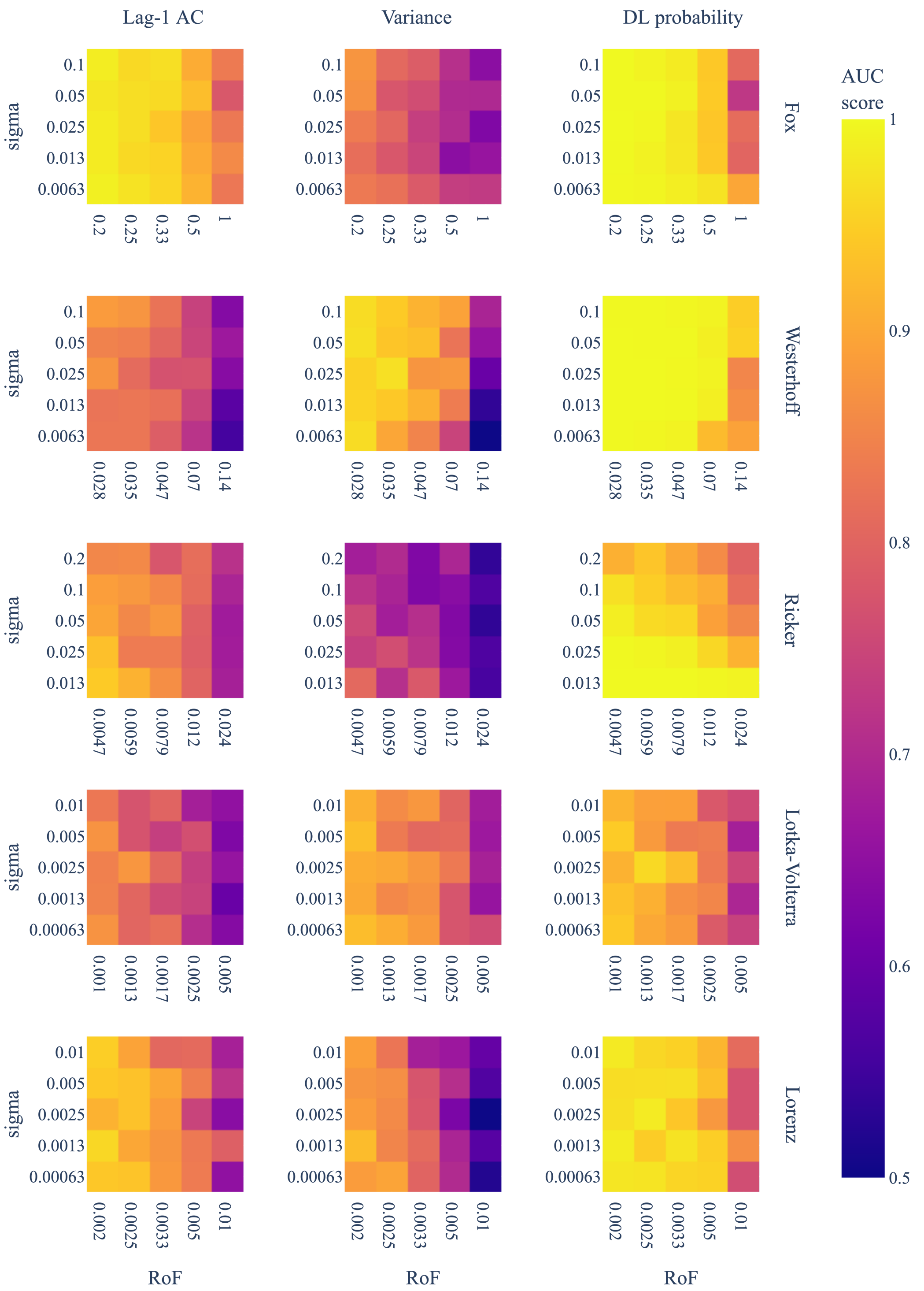

Figure S5: AUC score (area under the ROC curve) at different values of noise amplitude (sigma) and rate of forcing (RoF) for each indicator (columns) and theoretical model (rows). At each combination of RoF and sigma, we run 100 forced and null simulations, resulting in a total of 5000 simulations for each model. ROC curves are computed using predictions at 80% of the way through the pretransition time series. The bifurcations associated with each model are period-doubling (Fox), Neimark-Sacker (Westerhoff), fold (Ricker), transcritical (Lotka-Volterra), and pitchfork (Lorenz).

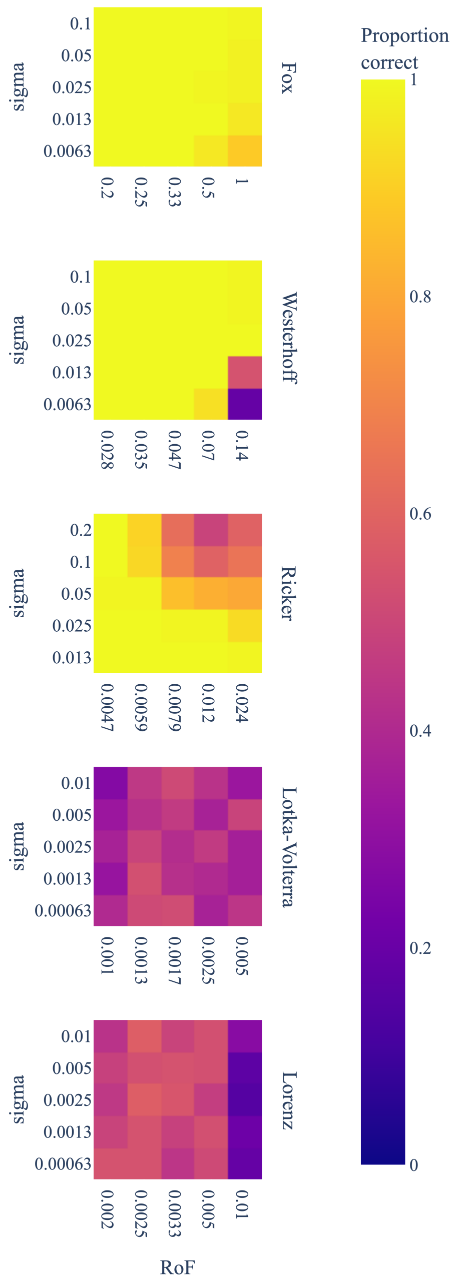

Figure S6: Proportion of predictions from the classifier that favoured the correct bifurcation for forced trajectories of different noise amplitude (sigma) and rate of forcing (RoF). For each combination of RoF and sigma, 100 forced trajectories are simulated, and predictions are made 80% of the way through the pretransition time series. The bifurcations associated with each model are period-doubling (Fox), Neimark-Sacker (Westerhoff), fold (Ricker), transcritical (Lotka-Volterra), and pitchfork (Lorenz).

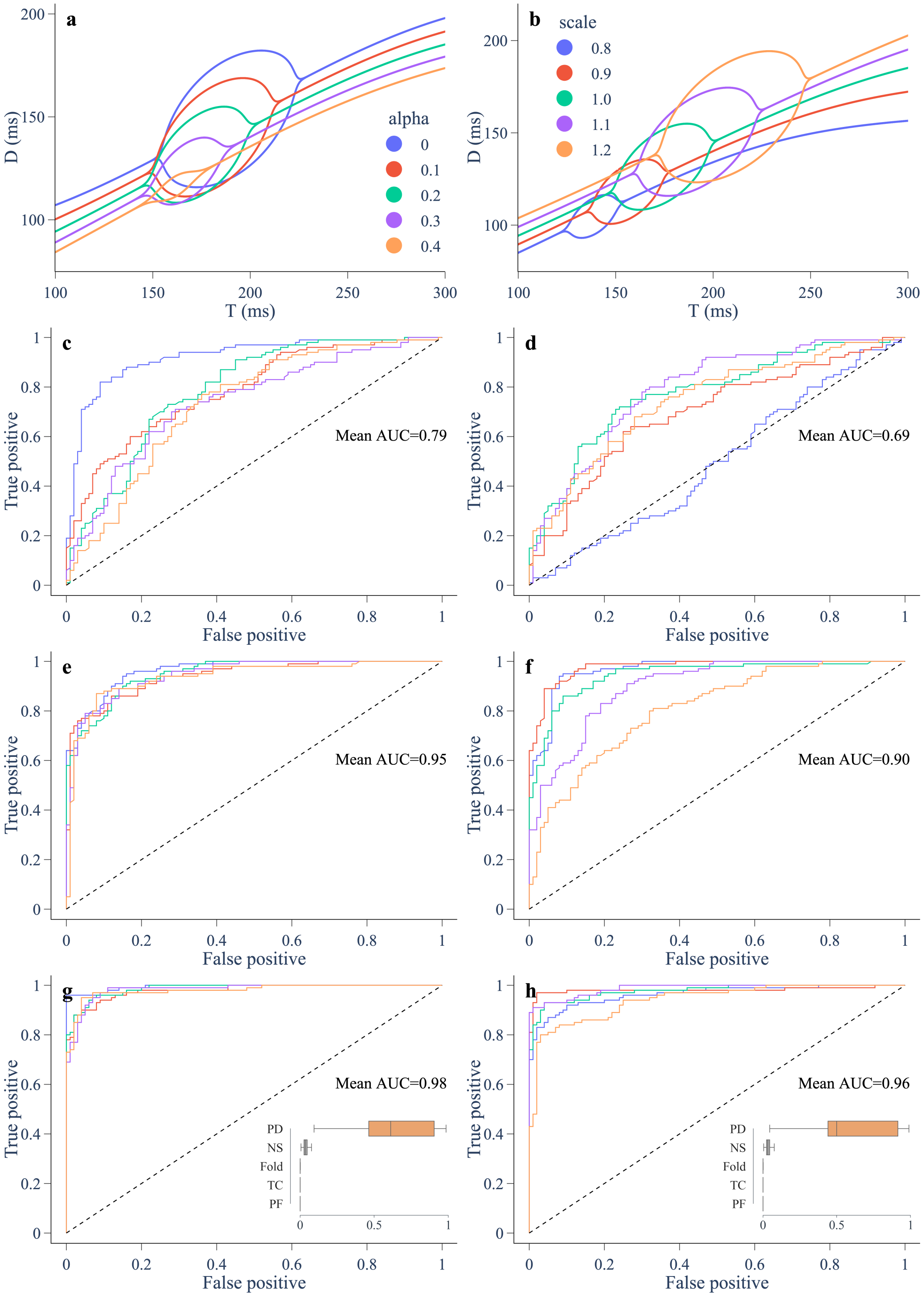

Figure S7: Performance of EWS in the Fox model for a range of model parameters. (a-b) Bifurcation diagrams for the Fox model at different values of alpha (influence of memory on action potential duration) and a multiplicative scaling of the restitution curve parameters. (c-d) ROC curves for variance. (e-f) ROC curves for lag-1 autocorrelation. (g-h) ROC curves for the DL classifier. Inset shows weight assigned by the DL classifier to each bifurcation. AUC is area under the ROC curve. Forced trajectories are simulated for 300 time steps with the bifurcation parameter T decreasing linearly from 300 to the bifurcation point and null trajectories are simulated with $T = 300$. Predictions are made 80% of the way through the time series.

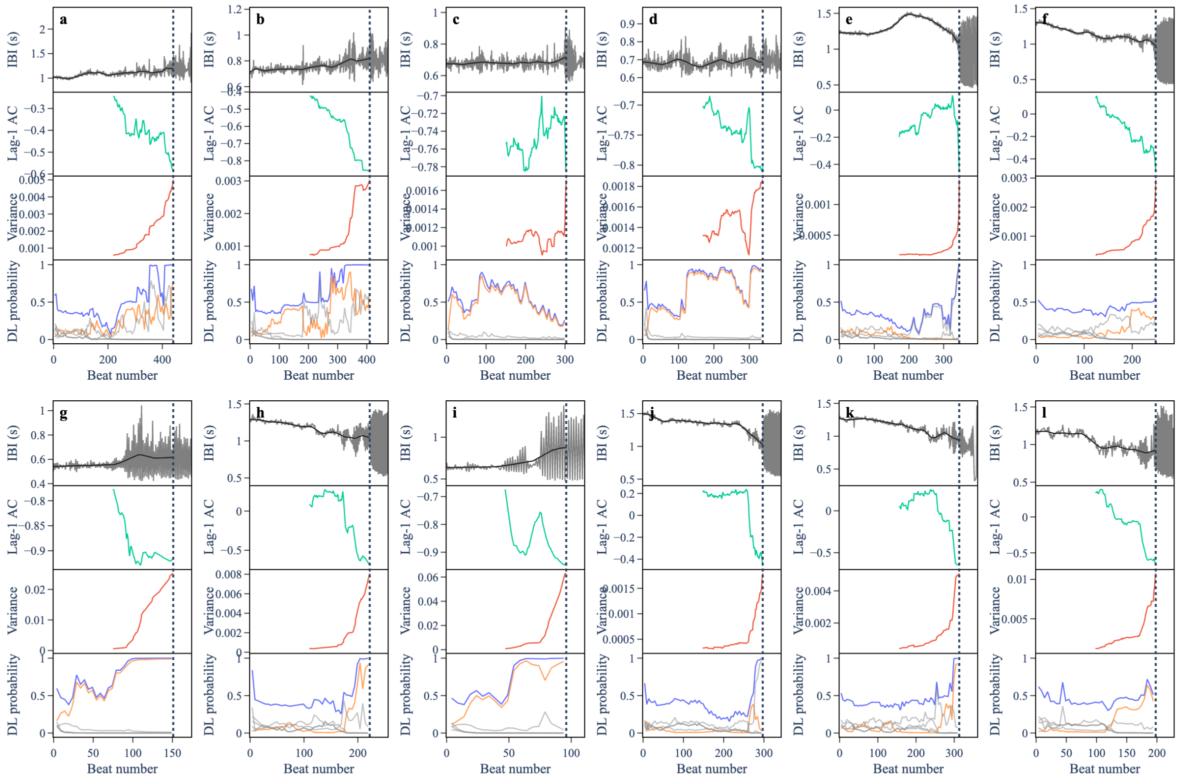

Figure S8: Trends in indicators prior to a period doubling bifurcation in chick heart aggregates (IDs 1-12). See Fig. 3 caption for details.

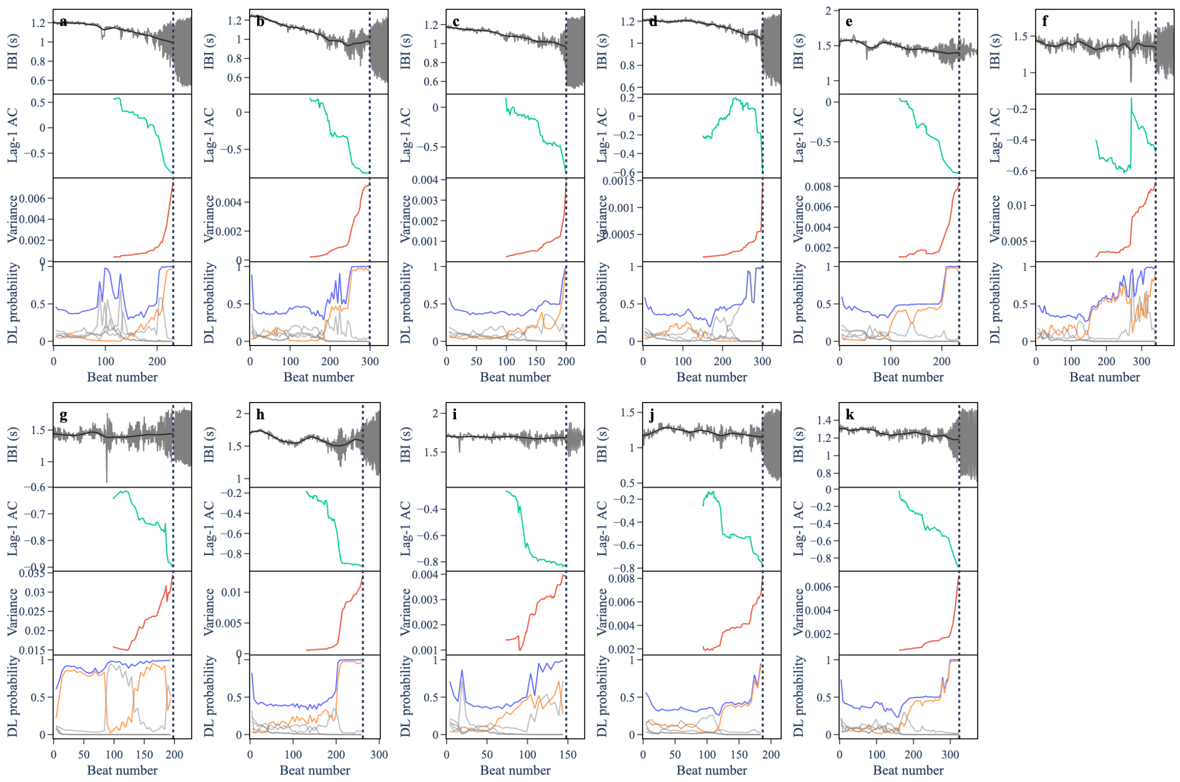

Figure S9: Trends in indicators prior to a period doubling bifurcation in chick heart aggregates (IDs 13-23). See Fig. 3 caption for details.

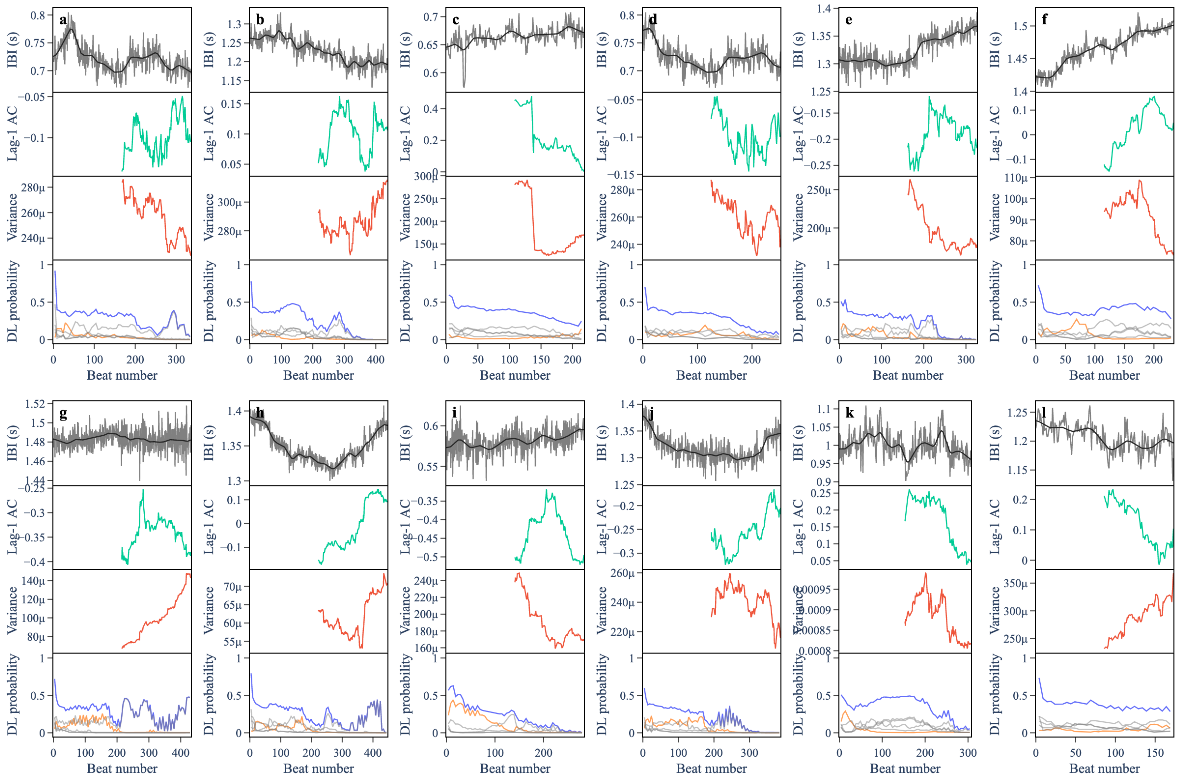

Figure S10: Trends in indicators for chick heart aggregates that do not undergo a period-doubling bifurcation (IDs 1-12). See Fig. 3 caption for details.

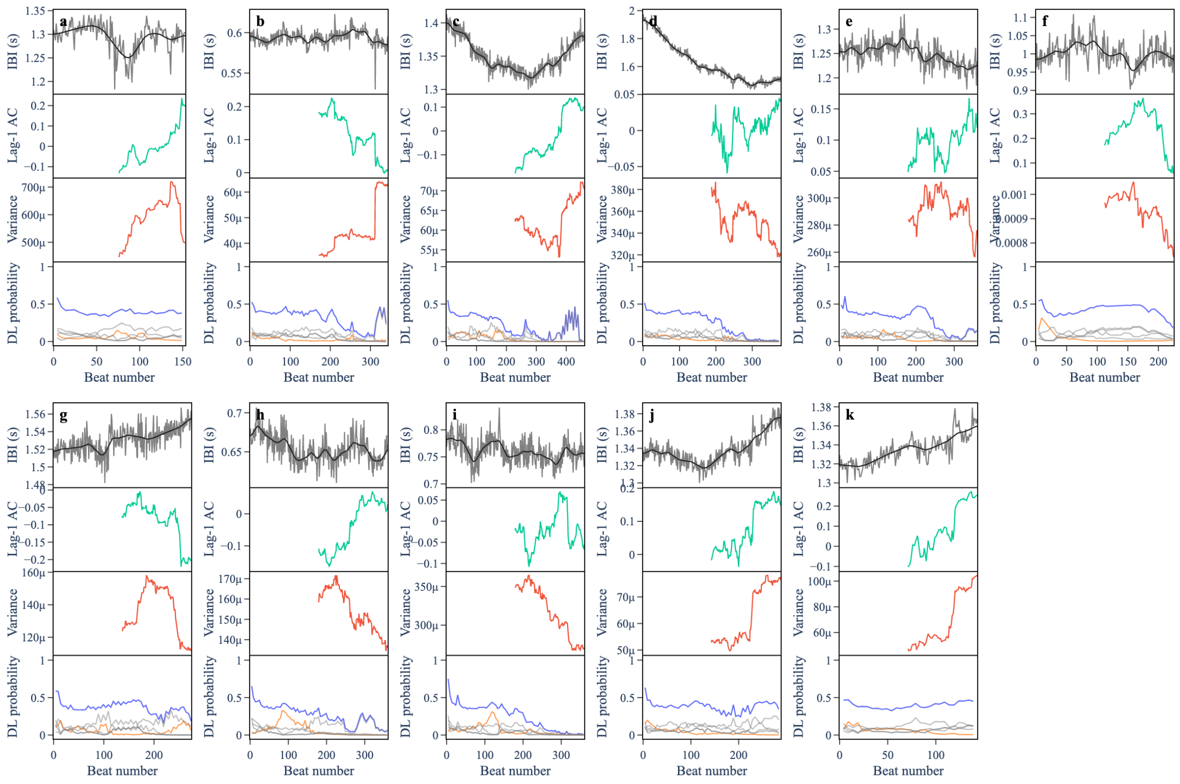

Figure S11: Trends in indicators for chick heart aggregates that do not undergo a period-doubling bifurcation (IDs 13-23). See Fig. 3 caption for details.

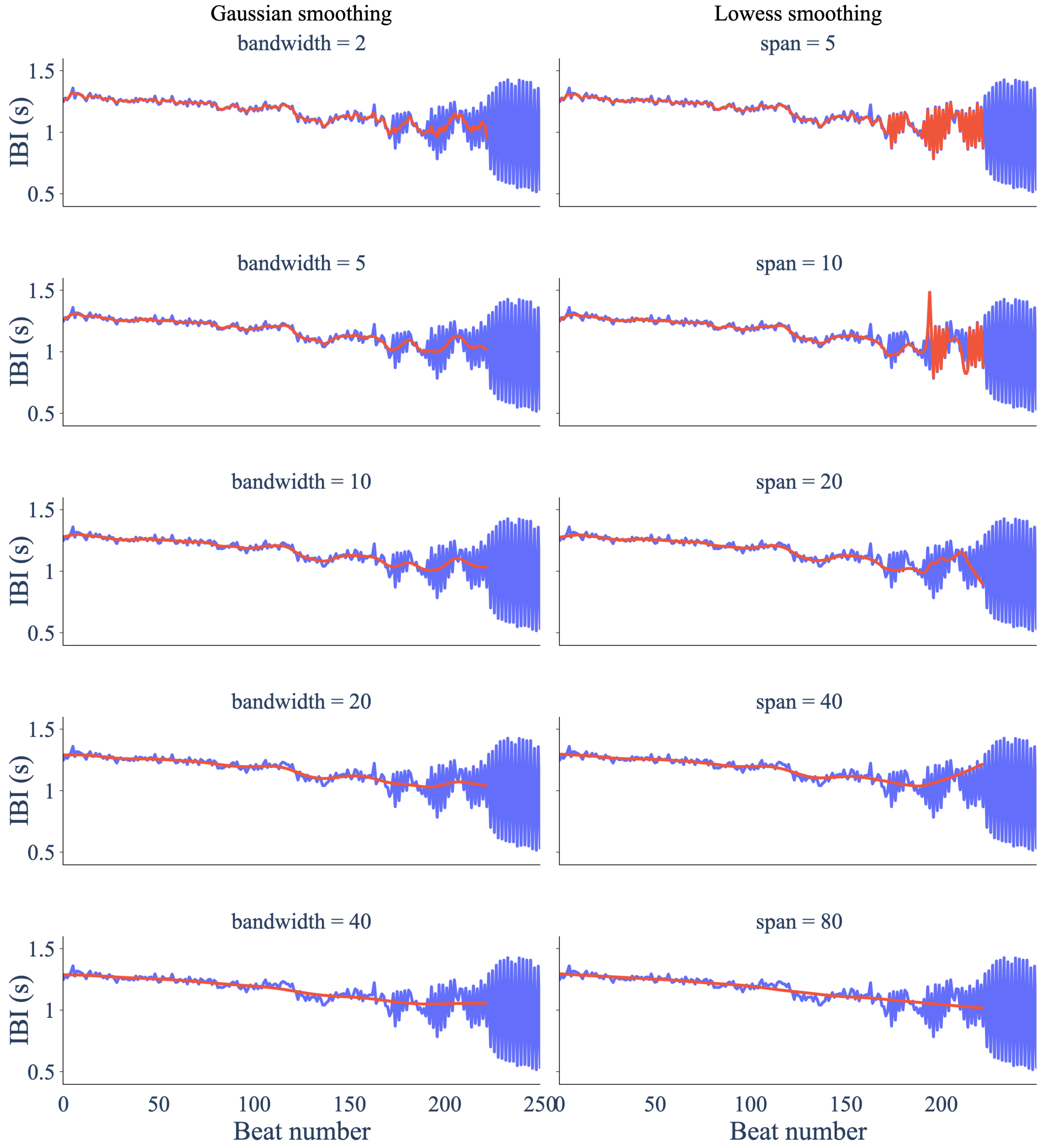

Figure S12: Detrending a sample record of the chick heart data (same as in Fig. 3a) using Gaussian smoothing (left) vs. Lowess smoothing (right) at different bandwidths and spans, respectively. Blue line shows chick heart data, red line shows smoothing. A bandwidth/span that is too small removes fluctuations that are relevant to the approaching bifurcation, whereas a bandwidth/span that is too large may not track trends in the equilibrium state. Performance of the EWS are tested using these smoothing configurations (Fig. S13). Results reported in the manuscript use Gaussian smoothing with a bandwidth of 20 beats.

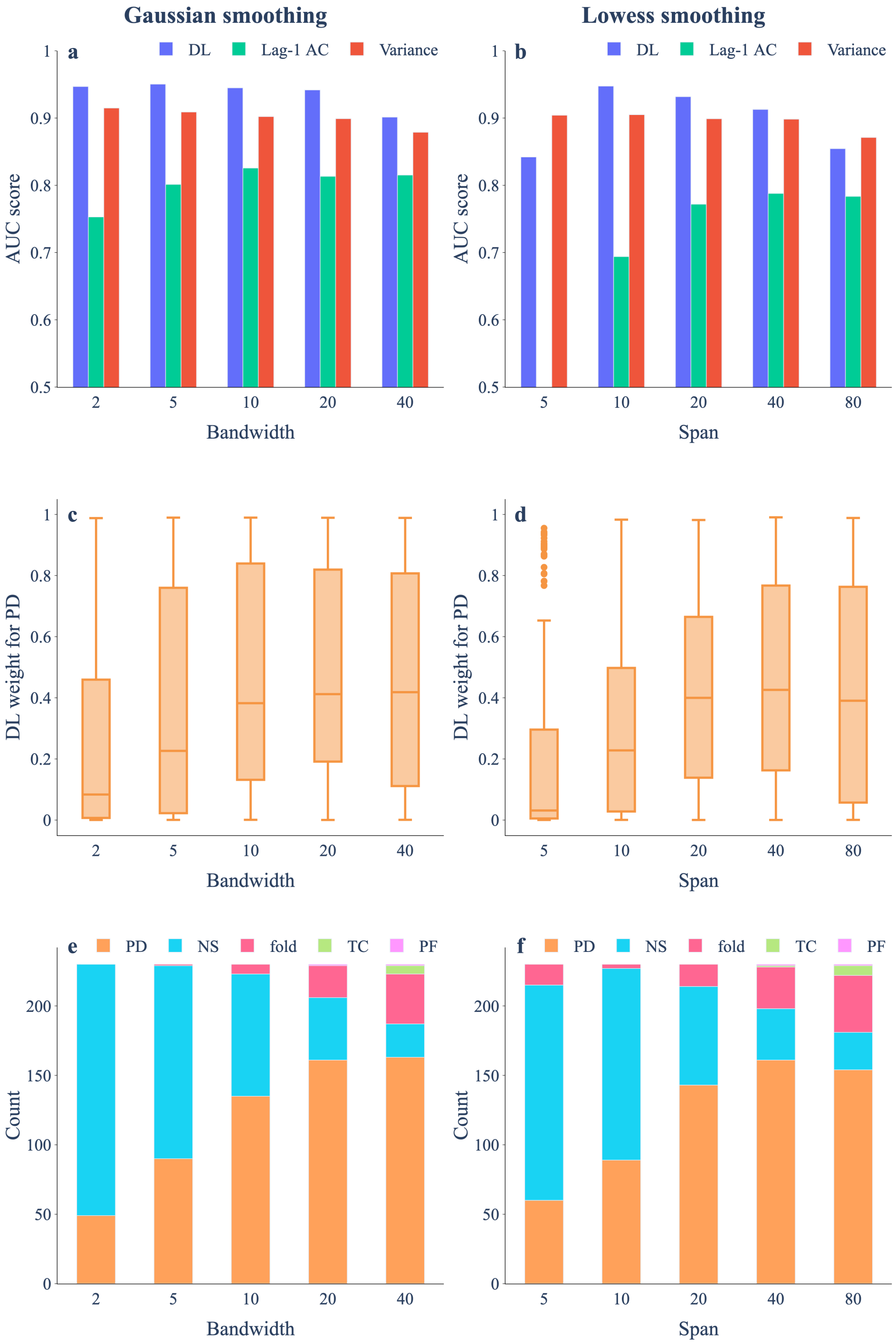

Figure S13: Performance of the DL classifier, lag-1 autocorrelation and variance on chick heart data using different detrending methods (Gaussian, Lowess) and parameters (bandwidth, span). (a-b) Area under the ROC curve (AUC) for each EWS. (c-d) DL weight assigned to the period-doubling bifurcation at the time of prediction. Box center line is the median, box limits are the upper and lower quartiles, whiskers mark the range up to 1.5 times the interquartile range, and points show outliers. (e-f) Bifurcation with the highest DL weight at time of prediction. Predictions are taken at 10 equally spaced time points between 60-100% of the way through the time series for each of the 46 records. PD: period-doubling, NS: Neimark-Sacker, TC: transcritical, PF: pitchfork.

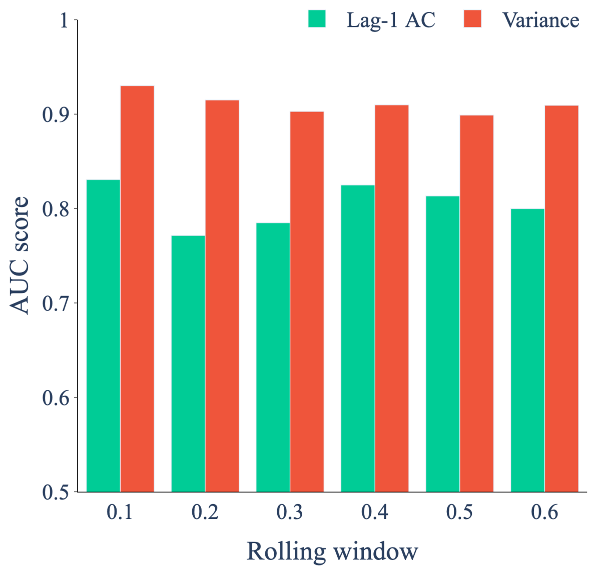

Figure S14: Performance of lag-1 autocorrelation and variance in the chick heart data using different sizes of rolling window. Time series are detrended using Gaussian smoothing with a bandwidth of 20 beats. Predictions are taken at 10 equally spaced time points between 60-100% of the way through the time series for each of the 46 records. AUC score: area under the ROC curve.

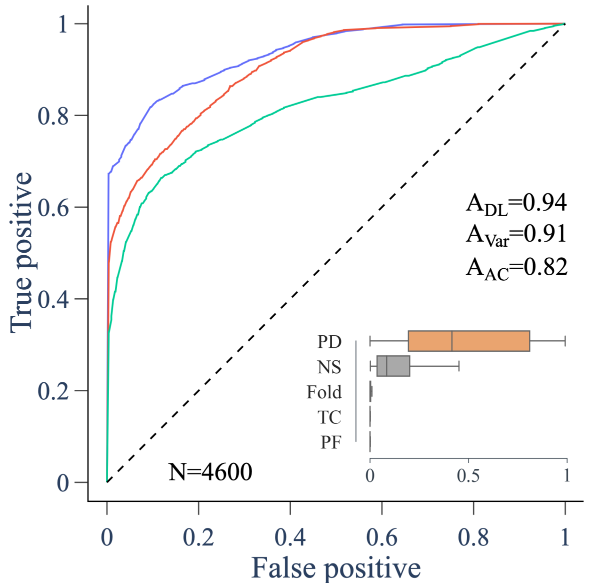

Figure S15: ROC curves for predictions of an upcoming transition in the chick heart data with a perturbation of size equal to the sampling error in the data. For each of the 23 neutral and period-doubling time series, 10 perturbed versions are created. Each data point is perturbed by a uniformly distributed random variable between -0.0125 and 0.0125. This results in a total of 4600 predictions. Other details in Fig. 4. PD: period-doubling. NS: Neimark-Sacker. TC: transcritical. PF: pitchfork.

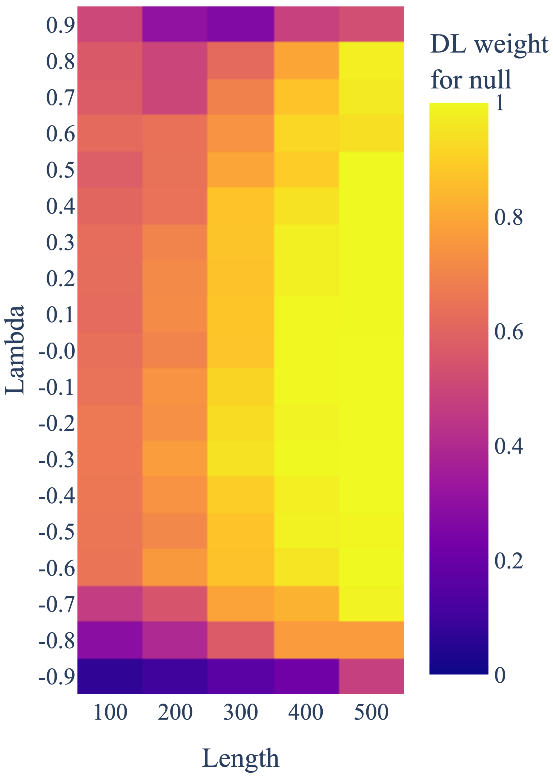

Figure S16: Mean weight assigned by DL classifier to the 'null' category for 100 simulations of the stationary AR1 process $y_{t+1} = \lambda y_t + \sigma\epsilon_t$ for different linear coefficient (λ) and length of simulation. Results are independent of σ since it acts only as a scaling factor. ϵ_t is drawn from the unit normal distribution. We find that the DL classifier is more confident identifying nulls in simulations that correspond to being far from a bifurcation ($|\lambda| << 1$) and that are longer in length.
